# Progressive cerebellar atrophy caused by heterozygous *TECPR2* mutations

**DOI:** 10.1002/mgg3.1857

**Published:** 2022-01-07

**Authors:** Keri Ramsey, Newell Belnap, Anna Bonfitto, Wayne Jepsen, Marcus Naymik, Meredith Sanchez‐Castillo, David W. Craig, Szabolcs Szelinger, Matthew J. Huentelman, Vinodh Narayanan, Sampath Rangasamy

**Affiliations:** ^1^ Center for Rare Childhood Disorders Translational Genomics Research Institute Phoenix Arizona USA; ^2^ Department of Translational Genomics Keck School of Medicine University of Southern California Los Angeles California USA

## CONFLICT OF INTEREST

The authors declare that there are no conflicts of interest.

To the Editor,

Biallelic mutations in *TECPR2* (OMIM #615000) are known to cause neurodevelopmental and progressive neurodegenerative disorders. TECPR2, an autophagy protein, regulates the lysosomal targeting of autophagosomes through interaction with ATG8 family proteins. A single *TECPR2* homozygous mutation was first identified as the cause of a novel subtype of hereditary spastic paraplegias (previously known as SPG49; OMIM #615031) in five individuals from three Jewish Bukharian families (Oz‐Levi et al., 2012). Through a recent international collaboration, Neuser et al. (2021) described the genotype and phenotype of an additional 17 individuals with bi‐allelic *TECPR2*‐variants, bringing the total count of affected individuals described in the literature up to 26 and expanding the phenotype to hereditary sensory and autonomic neuropathy type IX with developmental delay (HSAN9; OMIM #615031; Anazi et al., 2017; Heimer et al., 2016; Palma et al., 2021; Patwari et al., 2020; Zhu et al., 2015). The phenotype includes global developmental delay, intellectual disability, axial and appendicular hypotonia, dysarthria, spasticity, and ataxia. Peripheral neuropathy, areflexia and/or hyporeflexia, and autonomic dysfunction characterized by central hypoventilation and abnormal gastrointestinal motility are additional features. Here we describe for the first time a female patient diagnosed with progressive cerebellar atrophy with global developmental delay, hypotonia, areflexia, and sleep apnea with a stop‐gained mutation and a novel frameshift mutation in the *TECPR2* gene that resulted in loss of protein expression.

**FIGURE 1:**
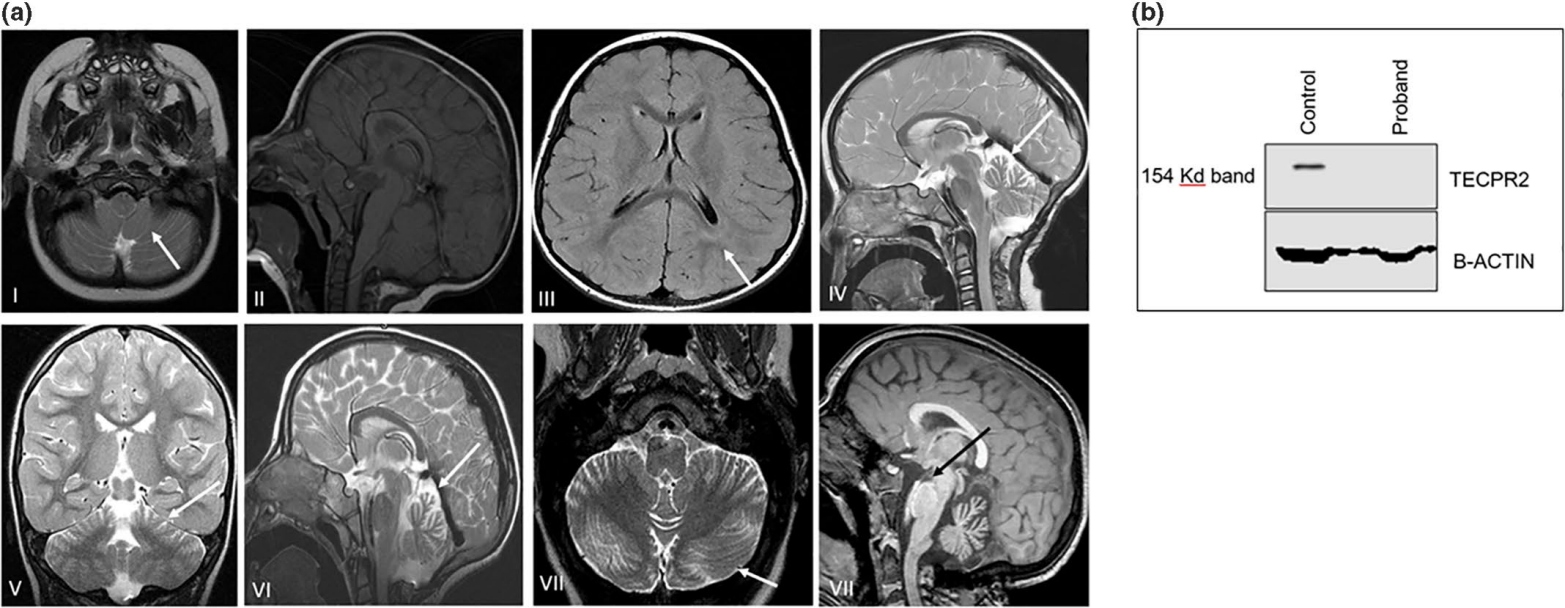
(a) MRI brain images (I) and (II) Chiari malformation type I at 14 months. (II) Normal CV at 14 months. (III) Delayed myelination at 3 years. (IV) Superior CV atrophy at 3 years. (V) and (VI) Progressive cerebellar atrophy (vermis and hemispheres) at 4 years. (VII) Worsening of cerebellar atrophy at 5 years 9 months. (VIII) Flattened pons at 5 years 9 months. (b) Western blot analysis of TECPR2 protein expression from skeletal muscle lysates. No observable band corresponding to the 154 kDa TECPR2 protein was defctected in the patient sample. CV, cerebellar vermis

The proband was a Caucasian female adopted at birth. As a newborn, she vomited frequently, had multiple bouts of pneumonia, and was diagnosed with failure to thrive. An MRI at five months showed a Chiari I malformation and tonsillar ectopia. At 17 months she underwent G‐tube insertion, Nissen fundoplication, and posterior fossa decompression. Over time her gait became more ataxic. She was jittery upon waking, wobbly, and had a tremor even upon standing. An MRI at this time showed atrophy of cerebellar vermis, olive, and pons while her myelination had improved (Figure 1a). A muscle biopsy at 3.5 years of age did not show specific histopathological features. Biochemical studies of the respiratory chain revealed normal enzyme activity. A repeat assay showed borderline complex I activity but was not sufficiently low on a consistent basis to be diagnostic itself. Between 3 and 5 years of age, she suffered progressive hearing loss, increased fatigue, worsening vision, and deterioration in speech with slurring. She still followed directions, crawled, and cruised. Clinical features included hypotonia, areflexia, facial weakness, a myopathic shape to her mouth, and midface hypoplasia. A sleep study revealed central sleep apnea, and she underwent a tonsillectomy and adenoidectomy. Over the last years of her life she became combative, aggressive, hurting herself and others, and with head banging when upset. She experienced episodes of posturing and left arm stiffness. An MRI of the brain at four years showed cerebellar vermis atrophy, flattening of the pons, and diffuse T2/FLAIR hyperintensity in the white matter (Figure 1a). She required supplemental oxygen. She passed away at age 5 years 10 months of hypoventilation due to central apnea.

The patient was deceased at the time of enrollment and the parents provided informed written consent for participation in the Western Institutional Review Board Protocol #20120789. Previously acquired DNA from blood and frozen skeletal muscle biopsy tissue from the vastus lateralis muscle was obtained.

Research whole‐exome sequencing analysis was performed on peripheral blood and revealed two heterozygous mutations in the *TECPR2* gene: (a) a novel dinucleotide deletion NM_014844.5:c.1318_1319del resulting in a frameshift mutation in exon 8, truncating the protein after 13 amino acids (p.Leu440fs) and (b) nonsense mutation NM_014844.5:c.3072G>A resulting in a stop codon (p.Trp1024Ter). The p.Trp1024Ter variant is listed in ClinVar (# 957432) as pathogenic by one commercial testing company and previously seen in an individual with autosomal recessive SPG49 (Landrum et al., 2018). Because biological parental samples were unavailable it is unclear whether the two variants are in *cis* or *trans*.

Truncated proteins due to loss of function (LoF) mutations, like the two described in our patient, are reported as being targeted for proteasome degradation (Oz‐Levi et al., 2012). We performed immunoblot analysis of skeletal muscle tissue lysate from the proband and a control sample to validate the functional consequences of both mutations. A TECPR2 band (140 kDa) was detected in the control human skeletal muscle sample but not in the patient skeletal muscle lysate (Figure 1b) suggesting that the truncated TECPR2 proteins are degraded. This finding supports our contention that the two *TECPR2* variants are *in trans*.

Mutations in *TECPR2* have been described in cases of autosomal recessive spastic paraplegia (SPG49) and classified under congenital disorders of autophagy, part of an emerging group of inborn errors of metabolism (Ebrahimi‐Fakhari et al., 2016). Research shows that TECPR2 is a human ATG8 binding protein and positive regulator of autophagy. Recent studies in fibroblasts of patients with *TECPR2* mutations have shown that the targeting of autophagosomes to lysosomes is performed through the TECPR2 carboxy‐terminal TECPR domain which interacts with VAMP8 (OMIM #603177), a lysosomal SNARE protein, as well as Atg8‐family proteins and the HOPS complex on the autophagosomal membrane through the TECPR2 LIR motif (Fraiberg et al., 2021). Tamim‐Yecheskel et al. (2021) created a *tecpr2* knockout mouse model demonstrating an age‐dependent accumulation of autophagosomes in the brain and spinal cord that suggest a defect in the targeting of these vesicles to the lysosomes. Stadel et al. (2015) described a role of TECPR2 in mediating COPII‐dependent endoplasmic reticulum export in cooperation with LC3C (OMIM #609605), a protein required for autophagosome formation. Depletion of TECPR2 reduced the numbers of ER exit sites (ERES) and substantially delayed ER export. HSP patient fibroblast cell lines with *TECPR2* mutations showed decreased levels of SEC24D (OMIM #607186), a COPII coat protein, and delayed ER export. These studies demonstrated that TECPR2 regulates autophagy, possibly through maintaining functional ERES.

Our patient showed the common features associated with TECPR2‐related disorder, including global developmental delay, intellectual disability, hypotonia, areflexia, dysarthria, and ataxia as well as central hypoventilation and delayed gastrointestinal motility. Her MRIs demonstrated progressive cerebellar atrophy, flattening of the pons, and early‐onset delayed myelination. As described in other individuals with *TECPR2* mutations (Patwari et al., 2020), our patient demonstrated progressive central apnea due to respiratory cycle dysregulation and eventually relied on supplemental oxygen and passed away from respiratory failure. Similar to other reported cases of HSAN9 our patient had two LoF mutations resulting in suspected proteasome degradation as demonstrated by western immunoblot analysis of her skeletal muscle tissue showing undetectable TECPR2 protein.

In conclusion, we report a child with a progressive, multisystem disorder who underwent exome sequencing using samples acquired post‐mortem. We suggest that she had a very rare disorder of autophagy caused by two truncating pathogenic variants in *TECPR2*. These findings further support the recent publication by Neuser et al. and demonstrate the severity of this form of neuropathy.

## Data Availability

The data that support the findings of this study are available on request from the corresponding author. The data are not publicly available due to privacy or ethical restrictions.
